# Toxic effects of AB-CHMINACA on liver and kidney and detection of its blood level in adult male mice

**DOI:** 10.1007/s11419-023-00670-0

**Published:** 2023-08-13

**Authors:** Soheir Ali Mohammad, Rasha Elhaddad Ali Mousa, Sahar Mohamed Gebril, Khaled Masoud Mohamed Masoud, Rania Ahmad Radwan

**Affiliations:** 1 Department of Forensic Medicine and Clinical Toxicology, Faculty of Medicine, Sohag University, Sohag, Egypt; 2 Department of Histology, Faculty of Medicine, Sohag University, Sohag, Egypt; 3 Department of Forensic Sciences, College of Criminal Justice, Naif Arab University for Security Sciences, Riyadh, Saudi Arabia

**Keywords:** LD_50_, AB-CHMINACA, Synthetic Cannabinoids SCs, Experimental Study, Histopathological Effects, GC–MS-MS

## Abstract

**Background:**

AB-CHMINACA is a cannabimimetic indazole derivative. In 2013, it was reported in different countries as a substance of abuse.

**Purpose:**

This study evaluated the subacute toxic effects of AB-CHMINACA on the liver and kidneys and measured its blood level in adult male mice.

**Methods:**

The histological and biochemical subacute toxic effects on the liver and kidneys were assessed after four weeks of daily intraperitoneal injections of one of the following doses: 0.3 mg/kg, 3 mg/kg, or 10 mg/kg as the highest dose in adult male albino mice. In addition, the blood concentration level of AB-CHMINACA was determined by GC–MS-MS.

**Results:**

The histological effects showed congestion, hemorrhage, degeneration, and cellular infiltration of the liver and kidney tissues. Considering the control groups as a reference, biochemical results indicated a significant increase in the serum AST only in the highest dose group, while the ALT and creatinine levels did not significantly change. The mean values of AB-CHMINACA blood levels were 3.05 ± 1.16, 15.08 ± 4.30, and 54.43 ± 8.70 ng/mL for the three treated groups, respectively, one hour after the last dose of intraperitoneal injection. The calibration curves were linear in the 2.5–500 ng/mL concentration range. The intra-assay precision and accuracy of the method were less than 7.0% (RSD) and ± 9.2% (Bias).

**Conclusion:**

This research supports the available case reports on AB-CHMINACA toxicity that it has low lethality; still, the chronic administration causes evident liver and kidney histotoxic effects even at low doses with unnoticeable clinical effects in mice.

## Introduction

AB-CHMINACA (*N*-[(2S)-1-amino-3-methyl-1-oxobutan-2-yl]-1-(cyclohexyl-methyl) indazole-3-carboxamide) is a third-generation synthetic cannabinoid (SC) which is an indazole carboxamide derivative containing L-Valinamide at the 3-carboxamide position [1]. In 2009, AB-CHMINACA was first synthesized as a potential medicinal drug, but in 2013, it was reported in different countries as a substance of abuse [2].

Cannabinoids exert their effects primarily through activating two cannabinoid receptors, cannabinoid receptor 1 (CB1) and cannabinoid receptor 2 (CB2) [3]. AB-CHMINACA has a high affinity to bind to those receptors and is estimated to be 11 to 58 times more potent than tetrahydrocannabinol (THC) in mice [4]. Numerous observational studies have reported symptoms of acute AB-CHMINACA toxicity, with neuropsychiatric symptoms being the most frequently reported. These included symptoms ranging from mild depression and disorientation to severe agitation, convulsions, and acute psychosis. These were followed by gastrointestinal symptoms and a wide range of cardiorespiratory presentations [5–7], and to a lesser extent, acute renal injury, fulminant liver failure, and stroke were observed [8–10].

Accurate estimation of the toxic and lethal doses of AB-CHMINACA for humans is challenging due to the nature of the commercial manufacturing method. The substance is usually dissolved in a vehicle and sprayed over herbs, making it unevenly distributed, which results in highly variable consumption levels of the illicit substance [11]. Most reported acute toxicity cases ended with complete recovery; death was uncommon [12]. Deaths from toxicity by SC compounds were either due to direct vital organ toxicity or severe CNS depression. In most cases, alcohol or other drugs of abuse were consumed concurrently, which may have augmented the toxic effects, or reduced the toxic dose of SCs [13].

Conventional THC detection kits cannot identify synthetic cannabinoids due to differences in their chemical structure, so sensitive and precise chromatographic techniques are favored, such as gas-chromatography mass spectrometry (GC–MS), high-performance liquid chromatography (HPLC) and liquid chromatography time of flight mass spectrometry (LCTOF-MS) [14, 15]. SCs levels can be detected in various biological samples, such as blood, urine, hair, and some tissues [16].

This study aimed to evaluate the subacute toxic effects of AB-CHMINACA on the liver and kidneys in adult male albino mice. A secondary aim was the detection of the AB-CHMINACA levels in the blood samples of treated mice in relation to different given doses.As the lethal dose of AB-CHMINACA was unknown, a preliminary experiment was done to detect the median lethal dose (LD_50_). LD_50_ was used as a guide for properly selecting doses used in the main experiment. In this study, we try to shed some light on the possible sublethal toxic effects of long-term drug administration that may impact chronic drug users and to understand better the acute toxic effects of SCs reported by various emergency units.

## Material and method

### Animals

Adult drug-naïve male Swiss albino mice weighing 30 ± 5 g were used; 12 mice for the LD_50_ experiment and 50 mice for the subacute toxicity experiment. The animals, a maximum of five mice per cage, were acclimatized to the lab conditions one week before experimentation in a controlled room temperature (22 ± 2 °C) and 12-h dark–light cycles with free access to water and pellet feed.

### Chemicals

A solid form of AB-CHMINACA was purchased from Cayman Chemicals, USA. The substance was dissolved in absolute ethanol followed by dilution in normal saline (ethanol: saline 1:9) immediately before injection. Human diagnostic kits for ALT and AST were from Roche Diagnostics USA.

### Instrument

Cobas C311 autoanalyzer (Roche Diagnostics, USA) was used for serum ALT, AST and creatinine measurement. Agilent GC–MS/MS model 7890B GC equipped with 7000C MS, Autosampler (Agilent 7693), and capillary column (HP-5MS 5% Phenyl Methyl Silox: 30 m x 250 µm, 0.25 µm) were used for detection and quantification of AB-CHMINACA in blood. A Leica DM500 microscope with an ICC50 camera system was used for histological examination.

### Method

#### The LD_50_ experiment

The LD_50_ was determined according to Lorke [17], which allows approximate LD_50_ detection using a limited number of animals in a two-step experiment. In the first step, nine mice were equally divided into three groups, each receiving intraperitoneal (IP) injection of AB-CHMINACA in a dose of 10, 100, or 1000 mg/kg. The animals were monitored continuously for eight hours, then every four hours for the remaining of the 1st 24 h, and daily for 14 days. Based on the number of deceased and living animals in the first step, further four doses (50, 100, 200, and 400 mg/kg) were chosen for the second step using a table proposed by Lorke. Each dose was given to a single mouse except for the 100 mg/kg dose which was already assessed in the first step. The animals were monitored using the same schedule as the first step, and then the LD_50_ was computed based on the dead-to-living animals ratio. LD_50_ was determined by taking the geometric mean of the two subsequent doses that showed 0% and 100% death (the highest nonlethal and the lowest lethal doses).

#### The subacute experiment

A total of fifty mice were allocated into five equal groups. Group A did not receive any treatment (negative control). Group B received the vehicle only (ethanol: saline) (positive control), while groups C, D, and E received daily IP AB-CHMINACA injections at doses of 0.3, 3, and 10 mg/kg/day, respectively, for four weeks. Clinical effects such as excitation and depression were recorded daily, and weekly body weights were taken. The animals were sacrificed under light anesthesia one hour after the last dose. Samples from jugular venous blood were collected for biochemical and toxicological analysis.

##### Biochemical analysis

Collected blood samples were centrifuged, and serum levels of AST, ALT, and creatinine were measured to assess liver and kidney functions.

##### Histopathological evaluation

Animals were dissected, and the liver and kidney were harvested and fixed in 10% neutral buffered formalin and transferred to 70% ethanol after two days. Tissues were processed, kept in paraffin blocks, and sectioned to a thickness of 4 µm. Hematoxylin and eosin (H&E) were used to stain the tissues before inspection under the light microscope.

##### Toxicological analysis

Whole blood samples were preserved with sodium fluoride (2 mg/mL) and potassium oxalate (2 mg/cc) as an anticoagulant and stored at -20 °C. Sample preparation started with adding 50 µL of Granisetron as internal standard (IS) (5.0 µg/mL) to a 200 µL blood sample. Acetonitrile (0.5 mL) was applied, vortexed for a minute, and centrifuged for 5 min. The supernatant was transferred to a 3-mL polypropylene tube with Phosphate buffer (2.5 mL, pH6) and then mixed for 1 min.

Extraction of samples was done by a C18 Solid-phase extraction (SPE) cartridge (CHROMABOND, Macherey–Nagel) [18]. The cartridge was conditioned by successively adding 3 mL of each; methanol, distilled water, and phosphate buffer (pH 6); the sample was left to flow under gravity. Washing was done by adding 3 mL of water followed by 3 mL of 5% acetonitrile. The cartridge was dried under a pressure of 20 psi for 20 min. 2 mL of freshly prepared dichloromethane: isopropanol (4:1) was used to separate AB-CHMINACA from the SPE cartridge. The solvent was evaporated by nitrogen steam at room temperature. Finally, samples were reconstituted by adding 50 µL of ethyl acetate and transferred to a GC vial for analysis. 4 µL was injected into the GC inlet.

**Fig. 1:**
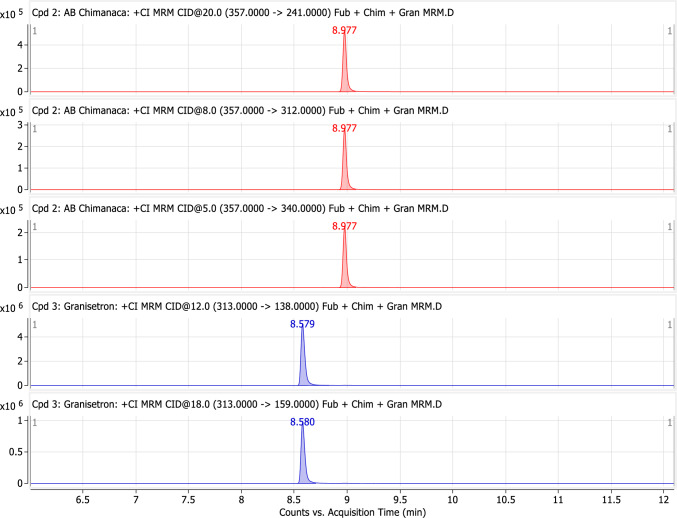
Multiple reactions monitoring chromatogram for AB-CHMINACA and Granisetron (IS) product ions. The red peaks represent the product ions of AB-CHMINACA (357- > 241, 357- > 312, and 357- > 340), and the blue peaks are for the product ions of the internal standard (313- > 138 and 313- > 159)

GC–MS-MS analysis was performed by helium as a carrier gas at a flow rate of 1 mL/min. The oven column temperature started at 200º C, held for 2 min, and then increased at a ramp of 20º C/min to 320º C, and then held for 6 min. The total run time was 12 min. Tandem mass spectrometry (MS/MS) was set to positive chemical ionization (PCI) mode. The ion source temperature was 300 °C, and the transfer line temperature was 280 °C. The used ionization gas was methane. The collision gas was argon. The multiple reaction monitoring (MRM) mode was applied to detect and quantify the compounds. MRM transitions and collision energies (CE) for AB-CHMINACA and IS are shown in Fig. 1.

The specificity, sensitivity, limit of detection (LOD), limit of quantification (LOQ), Linearity, precision, and accuracy were determined according to FDA (2018) guidelines [19]. Specificity of the method was evaluated by analysis of 7 blank blood collected from the negative control group. LOD and LOQ were calculated at signal-to-noise ratios (S/N) ≥ 3 and ≥ 10, respectively. Linearity of the method was determined by evaluation of the correlation coefficient (*r*^2^) for calibration curve. The calibration curve was prepared over the 2.5, 5, 25, 50, 100, and 500 ng/mL concentration range. The calibration curve was established by plotting the peak area ratios (analyte/IS) against the analyte concentrations. The intra-assay accuracy (% bias) and precision (% RSD) of the method was performed by analyzing six replicating of three quality control samples at concentrations of 7.5, 75, and 450 ng/mL. The values of acceptance for bias and RSD were ± 15% and 15%, respectively of the target compound.

### Statistical analysis

Data were analyzed using the Statistical Package for the Social Sciences “SPSS”software version 24. Normally distributed data were expressed as mean ± SD. Significance was tested by One-way ANOVA and post-Hoc tests at a P-value < 0.05. Data with skewed distribution were presented as median, minimum, and maximum. Significance was tested by Kruskal–Wallis H and Mann–Whitney U tests at a P-value < 0.05.

## Results

### Calculation of LD_50_

The numbers of dead animals in the first and second steps of the experiment are shown in Table 1. The calculated IP LD_50_ for AB-CHMINACA was 282.84 mg/kg.

**Table 1:** Results of Step 1 and Step 2 of the LD_50_ study

Dose	Number of dead over total number per dose
1st step (3 mice/group)	
10 mg/kg	0/3
100 mg/kg	1/3
1000 mg/kg	3/3
2nd step (single mouse/group)	
50 mg/kg	0/1
100 mg/kg*	1/3
200 mg/kg	0/1
400 mg/kg	1/1

*Result was taken from the 1st step

### Subacute toxicity

Mice from groups A, B, and C did not show any remarkable signs, while groups D and E demonstrated a short period of excitement followed by depression. The period of depression was brief in group D, about 15 min, and started to decrease gradually until complete disappearance on day ten. However, mice in group E showed a more extended period of depression, reaching about two hours in the experiment's early days, which started to slowly become briefer until reaching only 14 min on day 28. Certain animals in group E showed catalepsy, tachypnea or bradypnea, labored respiration, and generalized hair erection. There were no deaths except one mouse from group D, which died on the first day (mostly due to postural asphyxia secondary to CNS depression). Weekly weight measurements showed no statistically significant difference in animal weight across all groups, Table 2.

**Table 2:** Weight measurements in the studied groups

Statistics	Mean weight (g) ± SD				p-value
Group	Week 1	Week 2	Week 3	Week 4	
Group A	29 ± 3.6	30.6 ± 3.2	31 ± 2.6	32 ± 3.6	0.73
Group B	33.3 ± 0.6	32.6 ± 3.2	34.2 ± 5.3	33.3 ± 4.8	0.97
Group C	30.6 ± 2.9	29.8 ± 2.3	31.7 ± 1.9	31.5 ± 2	0.27
Group D	32.2 ± 4.3	31.3 ± 2.6	32.2 ± 1.9	31.7 ± 2.5	0.18
Group E	31.5 ± 2.8	28.7 ± 1.9	29.2 ± 2.1	29.6 ± 2.3	0.06

Biochemical analysis showed no significant differences in serum ALT across the five groups. However, serum AST was significantly higher in group E compared to the negative and positive control groups (p-value 0.034 and 0.004, respectively), with no significant differences between other study groups. As regards serum creatinine, there were no significant differences between the study groups, Table 3.

**Table 3:** The mean serum levels of ALT, AST and creatinine

Group (n = 10 in each group)	ALT^a^ (U/L)	AST^a^ (U/L)	Creatinine^b^ mg/dl
Group A (Negative control)	51.83 (± 20.29)	203.83 (± 40.94)	0.12 (0.10–0.33)
Group B (Positive control)	46.17 (± 8.23)	201.67 (± 22.91)	0.11 (0.09–0.28)
Group C (0.3 mg/kg)	53.43 (± 28.82)	256.71 (± 82.91)	0.12 (0.07–0.27)
Group D (3 mg/kg)	46.50 (± 10.71)	223.17 (± 41.76)	0.12 (0.10–0.28)
Group E (10 mg/kg)	63.33 (± 18.16)	282.83 (± 30.42)	0.16 (0.12–0.18)
p-value (Significant at the 0.05 level)	0.542	0.036*	0.184
A–B	0.998	1.000	0.683
A–C	1.000	0.754	0.346
A–D	0.999	0.990	0.869
A–E	0.954	0.034*	0.107
B–C	0.998	0.642	0.516
B–D	1.000	0.930	0.622
B–E	0.415	0.004*	0.171
C–D	0.999	0.973	0.189
C–E	0.994	0.992	0.057
D–E	0.482	0.145	0.086

^a^Results presented as mean ± standard deviation, p-value calculated by ANOVA and Post-Hoc test

^b^Results presented as median and minimum–maximum, p-value calculated by Kruskal–Wallis and Mann–Whitney U tests

^*^Significant

**Fig. 2:**
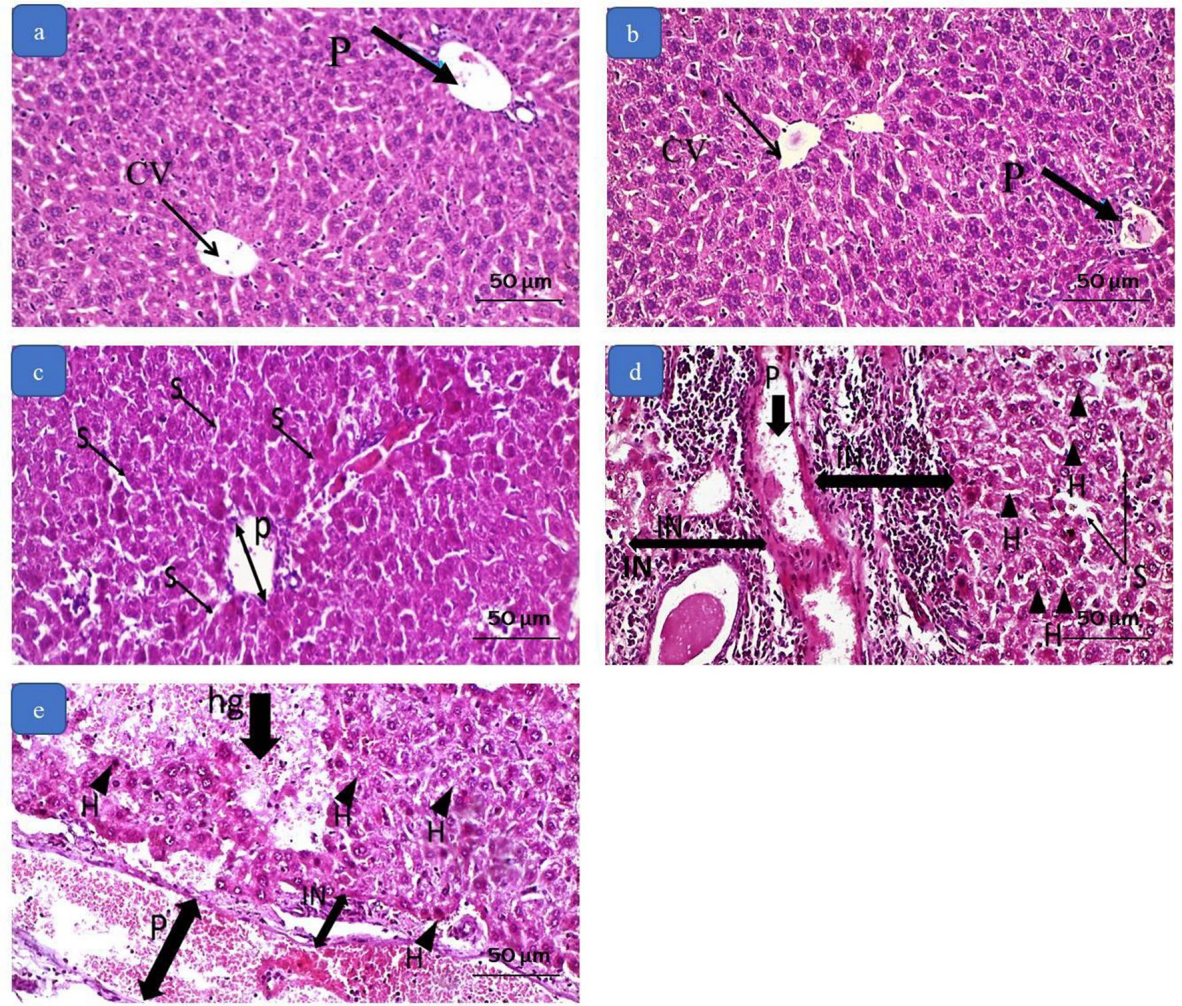
Photomicrographs of liver tissue by H&E. **a** and **b** section from the negative control and positive control group showing normal hepatocytes, central vein (CV), and portal vein (P). **c** section from group C (0.3 mg/kg) showing congested and dilated portal vein (P) and sinusoids (S). **d** section from group D (3 mg/kg) showing severely congested and dilated portal vein (P) and sinusoids (S), large foci of inflammatory cell infiltration (IN), and hepatocytes showing degeneration and apoptosis (H). **e** section from group E (10 mg/kg) showing severely congested and dilated portal vein (P), foci of inflammatory cell infiltration (IN), and hepatocytes showing degeneration and apoptosis (H). There is an area of interstitial hemorrhage (hg). (Magnification × 200)

The histopathological evaluation of both control groups showed a typical architecture of the hepatic lobules. The hepatic vessels and intrahepatic bile ducts were on average diameters, and the hepatocytes had a regular polyhedral shape with acidophilic cytoplasm and basophilic vesicular nuclei. On the other hand, liver sections from group C revealed mild dilatation and congestion of the central vein, portal vein, and blood sinusoids, and the hepatocytes showed hydropic degeneration. Sections from group D showed further dilatation and congestion of the hepatic vasculature with an accidental intraluminal thrombus, inflammatory cellular infiltration with foci of aggregation and hydropic degeneration, and apoptosis of the hepatocytes. In group E, the effects were considerably worse, where the liver architecture was disturbed with more significant vascular dilatation, congestion, and interstitial hemorrhage. Significant cellular infiltration and many inflammatory foci were present, and the liver cells showed different stages of degeneration and apoptosis, particularly around the portal vein, Fig. 2.

**Fig. 3:**
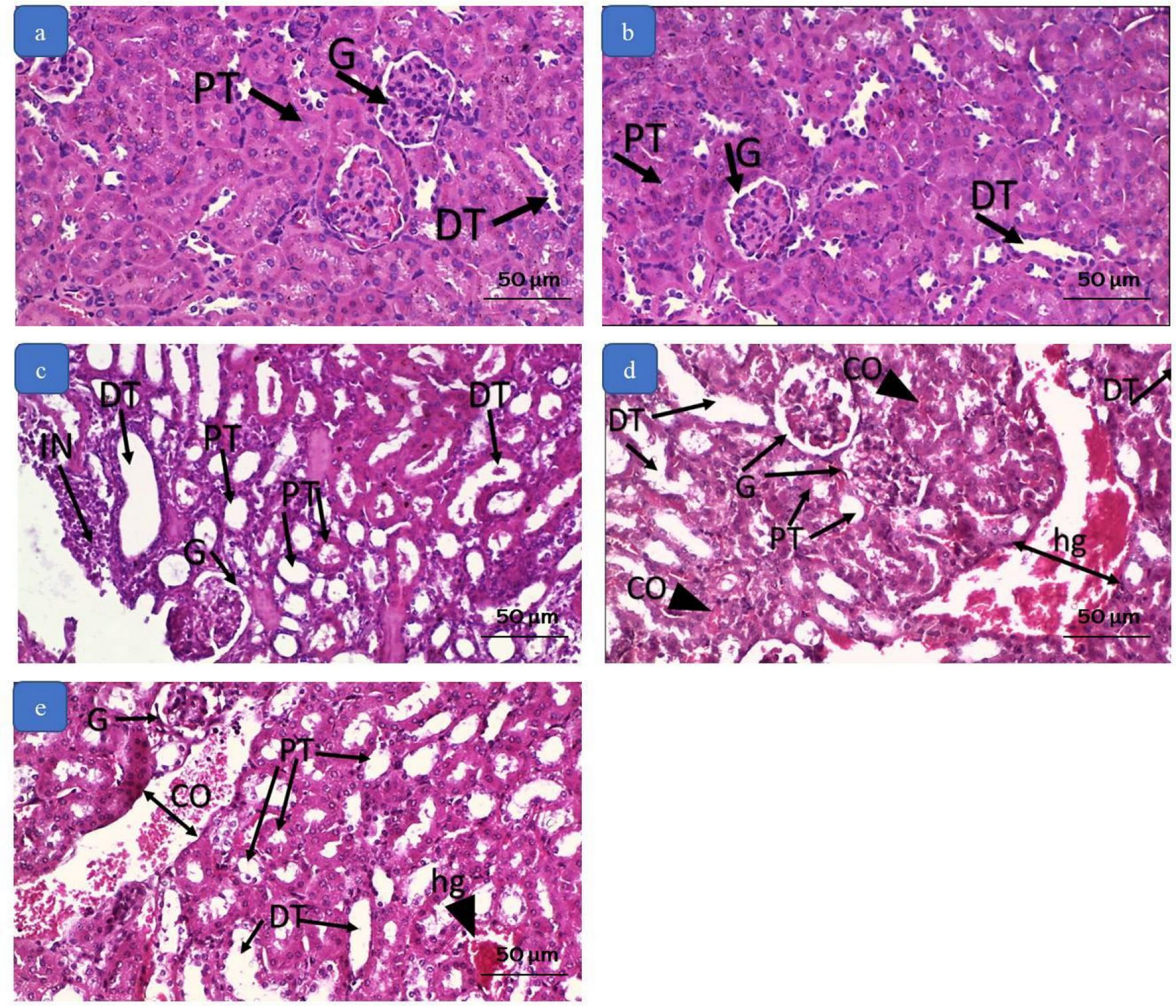
Photomicrographs of kidney tissue by H&E. **a** and **b** section from the negative control and positive control group showing normal proximal tubules (P.T.), distal tubules (D.T.) and renal glomeruli (G). **c**: section from group C (0.3 mg/kg) showing dilated proximal tubules (P.T.) and distal tubules (D.T.) with flattening of the lining epithelium. The renal glomeruli (G) are slightly affected. There is an area of inflammatory cell infiltration (IN). **d** section from group D (3 mg/kg) showing dilated proximal tubules (P.T.) and distal tubules (D.T.) with flattening of the lining epithelium. The renal glomeruli (G) show widened space. There are areas of hemorrhage (hg) and congestion (C.O.). **e** section from group E (10 mg/kg) showing dilated proximal tubules (P.T.) and distal tubules (D.T.) with flattening of the lining epithelium. The renal glomeruli (G) show widened space. There are areas of hemorrhage (hg) and congestion (C.O.). (Magnification × 200)

Examination of the kidney tissues in groups A and B showed preserved normal structure. The renal corpuscles and their capillary tufts, the proximal convoluted tubules, the distal convoluted tubules, the loop of Henle, and the collecting tubules were normal. Sections from group C showed dilated renal tubules with hydropic degeneration of the tubular epithelium in some tubules and flattening in others. The renal glomeruli were slightly affected, and the stroma was infiltrated by inflammatory cells. Group D showed expanded renal tubular lumens with flattened and degenerated epithelium, widened glomerular space, dilated and congested renal vasculature, and interstitial hemorrhage. Similar but more severe changes were also detected in group E, Fig. 3.

**Fig. 4:**
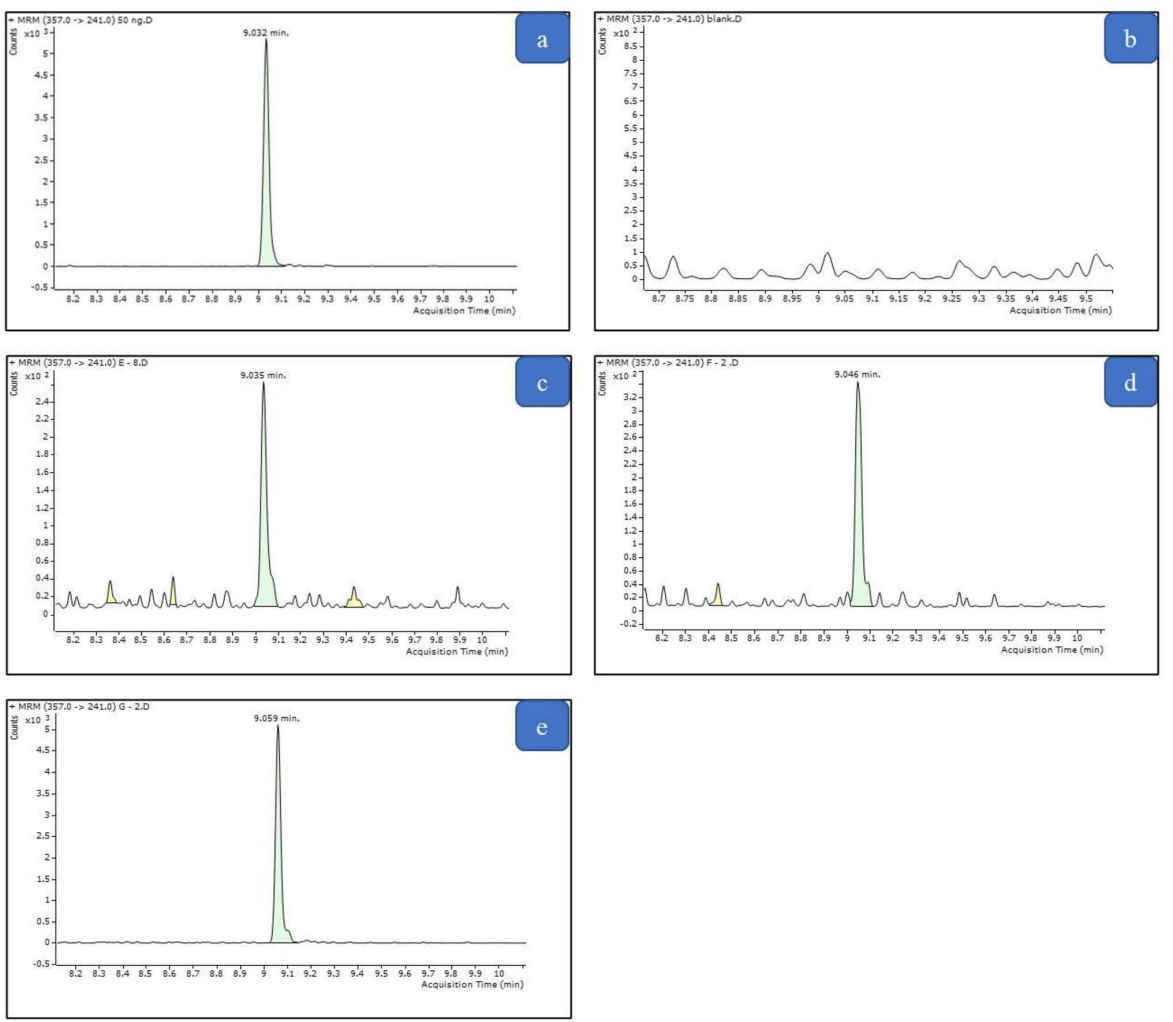
MRM chromatogram for the analysis of AB-CHMINACA in blood samples of the studied groups. **a** Spiked blood sample at 50 ng/mL of AB-CHMINACA. **b** Blank blood sample. **c** Blood sample from group C. **d** Blood sample from group D. **e** Blood sample from group E. The mean values of AB-CHMINACA levels in groups C, D, and E were 3.05 ± 1.16, 15.08 ± 4.30, and 54.43 ± 8.70 ng/mL, respectively

GC–MS-MS analysis showed no interfering peaks in blank samples at the retention times for AB-CHMINACA and IS, Fig. 4. The LOD and LOQ for AB-CHMINACA were 1.25 and 2.5 ng/mL, respectively. The calibration curve of AB-CHMINACA was linear over the range of 2.5–500 ng/mL (*r*^2^ > 0.99). Table 4 illustrates the values of accuracy and precision for AB-CHMINACA. As shown in the table, the values of RSD and bias were within the acceptable limit recommended by FDA (2018) guidelines.

**Table 4:** Precision and accuracy for AB-CHMINACA in mice blood

Replicates	QC1 7.5 ng/mL	QC2 75 ng/mL	QC3 450 ng/mL
1	8.47	75.94	514.21
2	7.74	86.24	469.53
3	8.17	74.85	483.29
4	8.43	84.85	475.39
5	7.61	76.31	507.58
6	8.01	73.54	498.23
Mean	8.07	78.62	491.37
SD	0.35	5.47	18.05
% RSD	4.39	6.95	3.67
% Bias	7.64	4.83	9.19

*SD* Standard deviation, *RSD* Relative standard deviation

The mean values of AB-CHMINACA level after one hour from the last IP injection in groups C, D, and E were 3.05 ± 1.16, 15.08 ± 4.30, and 54.43 ± 8.70 ng/mL, respectively.

## Discussion

SCs pose a public health hazard due to the lack of knowledge of their toxicity and unidentified negative health impacts and are often ingested in combination with other substances [20].

This study found that AB-CHMINACA has low lethality according to the toxicity classification of substances for IP administration by Berezovskaya [21], as the calculated LD_50_ was 282.84 mg/kg**.** Although there are no LD_50_ studies for AB-CHMINACA in the literature, our findings are comparable to those of studies on THC and other cannabinoids [22–24], even though the LD_50_ of a substance can vary depending on several variables, including the animal used, the route of administration, and the vehicle used [22, 25].

Although the lethal dose is high, the clinical effects of SCs appear at low doses. AB-CHMINACA (dose range 0.03 to 3 mg/kg, IP injection) showed complete substitution of THC and produced the tetrad response; motor depression, catalepsy, decreased pain sensation, and hypothermia in mice [4, 26, 27] which was similar to our results. In our experiment, the duration of action was notably short, which can be explained by the rapid metabolism of AB-CHMINACA [28], and the period of depression had much decreased with repeated injections, suggesting the development of tolerance as described with the synthetic cannabinoid AB-FUBINACA [29, 30]. Drug tolerance in our experiment could also be due to the concurrent use of ethanol which may have led to decreased sensitivity to SC [31], a common combination used by addicts.

The present study showed no effect of AB-CHMINCA on mice's body weight, reflecting no impact on the appetite. Interestingly, previous reports by De Vry et al. [32], Williams and Kirkham [33], Freedland et al. [34], Foltin et al. [35], and Beal et al. [36] showed that THC and its derivatives increase appetite in both humans and animals while cannabinoid antagonists are appetite suppressants. The different results could be attributed to the varying routes of administration, as reported by Manwell et al. [37], who found that vapored THC was associated with increased food intake in rats, while IP administration of THC did not affect food consumption.

The liver is the primary site of most exogenous and endogenous compounds metabolism; hence, it is a potential target for xenobiotic toxicity [38]. Cases of liver toxicity following a history of SCs consumption have been reported, which were diagnosed by elevated ALT and AST, bilirubin level, alkaline phosphatase, and INR. Variable degrees of liver toxicity were reported; however, most of those patients eventually improved, which suggests that the liver damage was at least partially reversible [39, 40].

The available literature did not have any histopathological evaluation of the chronic toxicity of AB-CHMINACA. However, the histopathological results of the present study are close to those reported in acute toxicity studies of other SCs, as mentioned by Abass et al. [23] and Bakdash et al. [24], who explored the LD_50_ of voodoo extract and THJ-2201 in rodents. Similarly, Abdelmoneim et al. [41] mentioned comparable results after chronic oral administration of Strox in Rats. The biochemical assay in the present study showed an increase in serum AST in the higher-dose group and no change in serum ALT which can be explained by injecting low doses of AB-CHMINACA and the liver's capacity to compensate for damage to an extent to preserve its function. Furthermore, AST is elevated not only by liver damage but by other organ affection, such as the heart and muscles, which is expected to be present [41–43].

Most substances are urinary excreted, so it is crucial to evaluate the toxic effects of any drug on the renal tissue [44]. Bhanushali et al. [45], Buser et al. [46], and Gudsoorkar and Perez [47], who supported our results, reported symptoms of acute kidney injury and renal failure in many cases after consumption of different types of SCs with elevated serum urea, creatinine and BUN, while urine analysis was positive for proteins and casts. Examination of the renal biopsy showed dilated renal tubules with epithelial degeneration and crystals inside the lumen and inflammatory cell infiltration in the surrounding stroma. Also, Abbas et al. [23] and Bakdash et al. [24], who explored the LD_50_ of THJ-2201 and voodoo extract, showed similar histopathological changes in the kidney.

Tissue damage caused by AB-CHMINACA could be attributed to the oxidative stress caused by the direct effect on mitochondrial respiratory enzymes with the decrease in the rate of O_2_ consumption and increase in the levels of hydrogen peroxide [48]. In addition to the direct toxic effect, nephrotoxicity in some cases could be due to rhabdomyolysis from convulsion and agitation [49].

This study proposed a GC–MS-MS detection method that could detect AB-CHMINACA in whole blood samples of the treated animals, one hour after the last dose of repeated IP injection. Some researchers found it possible to identify the parent drug in postmortem human blood samples [28, 50], while others could not [51]. The different results could be attributed to many factors, including the dose of exposure (which is difficult to be calculated in abusers due to the manufacturing process), the frequency of exposure, and the time between exposure and sampling, which is lacking in most studies. It is known that AB-CHMINACA is rapidly metabolized in the body [28], although there is no information about how long the parent drug would take to disappear from blood samples.

## Conclusion and recommendations

AB-CHMINACA is a synthetic cannabinoid substance of low lethality with LD_50_ of 282.84 mg/kg via the IP route. The central nervous system and respiratory systems were the most obviously clinically affected. There were evident histotoxic effects on the liver and kidney even at low dosages without clinically significant consequences, and these effects increased with exposure time and dose.

The proposed GC–MS-MS method for detecting AB-CHMINACA in blood samples was successfully applied to mice blood samples in the present study. However, further research is recommended to find the parent compound blood levels at different time intervals and correlate them to the levels of its major metabolites to establish the detection window for AB-CHMINACA as it is rapidly metabolized.
